# Redistribution of Cerebral Blood Flow during Severe Hypovolemia and Reperfusion in a Sheep Model: Critical Role of α1-Adrenergic Signaling

**DOI:** 10.3390/ijms18051031

**Published:** 2017-05-11

**Authors:** René Schiffner, Sabine Juliane Bischoff, Thomas Lehmann, Florian Rakers, Sven Rupprecht, Juliane Reiche, Georg Matziolis, Harald Schubert, Matthias Schwab, Otmar Huber, Martin Schmidt

**Affiliations:** 1 Orthopedic Department, Campus Eisenberg, Jena University Hospital-Friedrich Schiller University, 07607 Eisenberg, Germany; G.Matziolis@krankenhaus-eisenberg.de; 2 Department of Neurology; Jena University Hospital-Friedrich Schiller University, 07743 Jena, Germany; Florian.Rakers@med.uni-jena.de (F.R.); Sven.Rupprecht@med.uni-jena.de (S.R.); Matthias.Schwab@med.uni-jena.de (M.S.); 3 Institute for Laboratory Animal Sciences and Welfare, Jena University Hospital-Friedrich Schiller University, 07743 Jena, Germany; Sabine.Bischoff@med.uni-jena.de (S.J.B.); harald.schubert@outlook.de (H.S.); 4 Institute of Medical Statistics, Computer Sciences and Documentation Science, Jena University Hospital-Friedrich Schiller University, 07743 Jena, Germany; thomas.lehmann@med.uni-jena.de; 5 Institute for Biochemistry II, Jena University Hospital-Friedrich Schiller University, 07743 Jena, Germany; Juliane.Reiche@med.uni-jena.de (J.R.); Otmar.Huber@med.uni-jena.de (O.H.); Martin.Schmidt@med.uni-jena.de (M.S.)

**Keywords:** adrenergic regulation, alpha-adrenergic, cerebral blood flow, cerebral hemodynamics, resuscitation, head trauma, cerebrovasvular disease, neurodegenerative disease

## Abstract

Background: Maintenance of brain circulation during shock is sufficient to prevent subcortical injury but the cerebral cortex is not spared. This suggests area-specific regulation of cerebral blood flow (CBF) during hemorrhage. Methods: Cortical and subcortical CBF were continuously measured during blood loss (≤50%) and subsequent reperfusion using laser Doppler flowmetry. Blood gases, mean arterial blood pressure (MABP), heart rate and renal blood flow were also monitored. Urapidil was used for α1A-adrenergic receptor blockade in dosages, which did not modify the MABP-response to blood loss. Western blot and quantitative reverse transcription polymerase chain reactions were used to determine adrenergic receptor expression in brain arterioles. Results: During hypovolemia subcortical CBF was maintained at 81 ± 6% of baseline, whereas cortical CBF decreased to 40 ± 4% (*p* < 0.001). Reperfusion led to peak CBFs of about 70% above baseline in both brain regions. α1A-Adrenergic blockade massively reduced subcortical CBF during hemorrhage and reperfusion, and prevented hyperperfusion during reperfusion in the cortex. α1A-mRNA expression was significantly higher in the cortex, whereas α1D-mRNA expression was higher in the subcortex (*p* < 0.001). Conclusions: α1-Adrenergic receptors are critical for perfusion redistribution: activity of the α1A-receptor subtype is a prerequisite for redistribution of CBF, whereas the α1D-receptor subtype may determine the magnitude of redistribution responses.

## 1. Introduction

Severe bleeding (hemorrhage) is a common cause of disability following traumatic ruptures of large vessels and parenchymal organs, gastrointestinal bleeding or placenta abruption [1,2,3,4,5]. The sub-population most at risk for severe trauma and hemorrhage are people under 45 years [1]. The long-term functional outcome is largely determined by the degree of brain damage resulting from shock-associated cerebral hypoperfusion [6,7]. Although redistribution mechanisms maintain cerebral perfusion constant over a wide range of reduced blood pressures [8], this centralization of the circulation under shock conditions does not effectively prevent from cerebral injury [9]. The cerebral cortex appears to be the most vulnerable brain structure under severe shock conditions [7,9]. The origin of this vulnerability remains unclear. It is generally attributed to (1) high susceptibility of cortical neurons to ischemia or (2) to insufficient maintenance of cortical perfusion compared to subcortical structures [10,11,12]. Resolving this issue will be of crucial importance for clinical medicine since it may lead to development of new strategies for attenuating cortical damage during shock. Here we tested the hypothesis that the effectiveness of cerebral redistribution mechanisms under clinically relevant conditions (hemorrhagic shock) is inferior in the phylogenetic younger cerebral cortex than in the phylogenetically older subcortex, which contains vital regions such as the respiration and circulation control centers. The plausibility of this hypothesis is supported by some evidence for differences in cerebral blood supply in different brain regions [13]. We used a sheep model of hypovolemia since several features of this animal model like body weight, blood volume, cerebral gyration and cerebral vascular supply are similar to humans. Importantly, we continuously measured cerebral blood flow (CBF) during blood loss and reperfusion in the cortex and subcortical areas. In contrast, CBF has been monitored discontinuously in previous studies using experimental shock models [14,15,16], an approach that has possibly led to gaps in the understanding of cerebral perfusion maintenance during hypovolemia.

We further considered the possibility that regional differences in α1-adrenergic receptor densities exist and that these differences enable region-specific maintenance of cerebral perfusion during severe hemorrhage. Evidence for different levels of α1-adrenergic receptor expression in brain blood vessels have not been presented up to now (to our knowledge). However, two general considerations seemed to justify our adrenergic hypothesis. First, the α1-adrenergic system is the major vasoconstrictory effector of sympathetic activation during hemorrhagic shock [17]. And second, a lower concentration of α1-adrenergic receptors in the cerebral than in the peripheral circulation prevents from cerebral vasoconstriction during severe hemorrhage and is critically involved in cerebral blood supply [18,19]. We tested the role of the α1-adrenergic system by applying the specific blocker urapidil and continuously measuring CBF during subsequent blood loss and reperfusion.

## 2. Results

### 2.1. Effects of Severe Hemorrhage on Arterial Blood Gases and Vital Parameters

**Figure 1:**
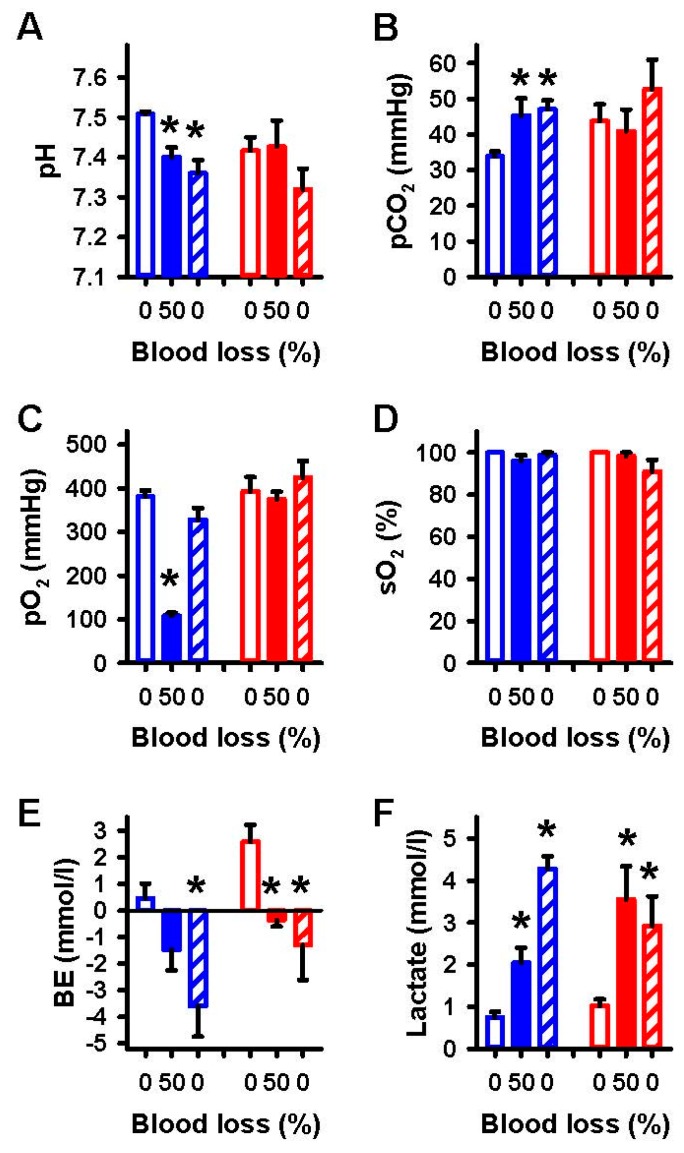
Effects of 50% blood loss and reperfusion on arterial blood gases and lactate. Values are given for baseline (open bars), removal of 50% blood (filled bars) and complete reperfusion (hatched bars) in controls (blue) and after α1A-adrenergic blockade (red) for (**A**) pH, (**B**) partial pressure of carbon dioxide (pCO_2_), (**C**) partial pressure of oxygen (pO_2_), (**D**) oxygen saturation (sO_2_), (**E**) base excess (BE) and (**F**) lactate. Mean ± SEM; * *p* < 0.05 compared to baseline.

Arterial blood gases and lactate were within their physiological ranges before the onset of controlled hemorrhage and after reperfusion (Figure 1). Hemorrhage induced a decrease of pH and pO_2_, and an increase of pCO_2_ and lactate (*p* < 0.05, Figure 1). Reperfusion could not normalize pH and pCO_2_, whereas pO_2_ was restored to baseline after reperfusion. The increase of lactate and the decrease of base excess were exaggerated after reperfusion. Oxygen saturation was unchanged during the course of the experiments.

**Figure 2:**
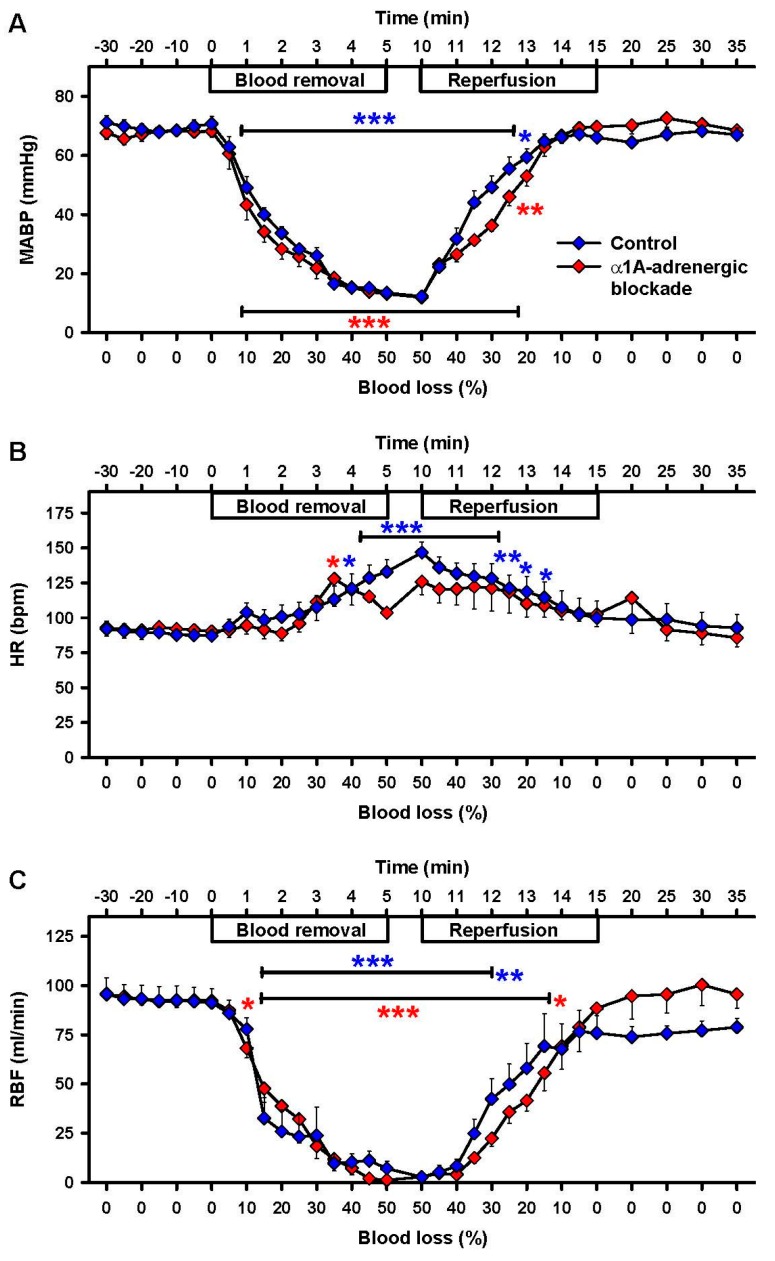
Effects of 50% blood loss and reperfusion on vital parameters. (**A**) Mean arterial blood pressure (MABP), (**B**) heart rate (HR) and (**C**) renal blood flow (RBF). Controls in blue and α1A-adrenergic blockade in red. Mean ± SEM; * *p* < 0.05, ** *p* < 0.01 and *** *p* < 0.001 compared to baseline.

Fifty percent loss of blood volume induced a decrease in mean arterial blood pressure (MABP) from 71 ± 3 mmHg to 14 ± 1 mmHg (*p* < 0.001), an increase of heart rate (HR) from 87 ± 5 bpm to 133 ± 9 bpm (*p* < 0.001) and a decrease in renal blood flow (RBF) from 91 ± 7 mL/min to 3 ± 2 mL/min (*p* = 0.008; Figure 2). Reperfusion restored the MABP to 68 ± 2 mmHg and the HR to 93 ± 10 bpm, neither of which were different from baseline values at the end of the recovery period (Figure 2). The RBF reached 79 ± 4 mL/min, still 14% below baseline.

### 2.2. Effects of Severe Hemorrhage on CBF

**Figure 3:**
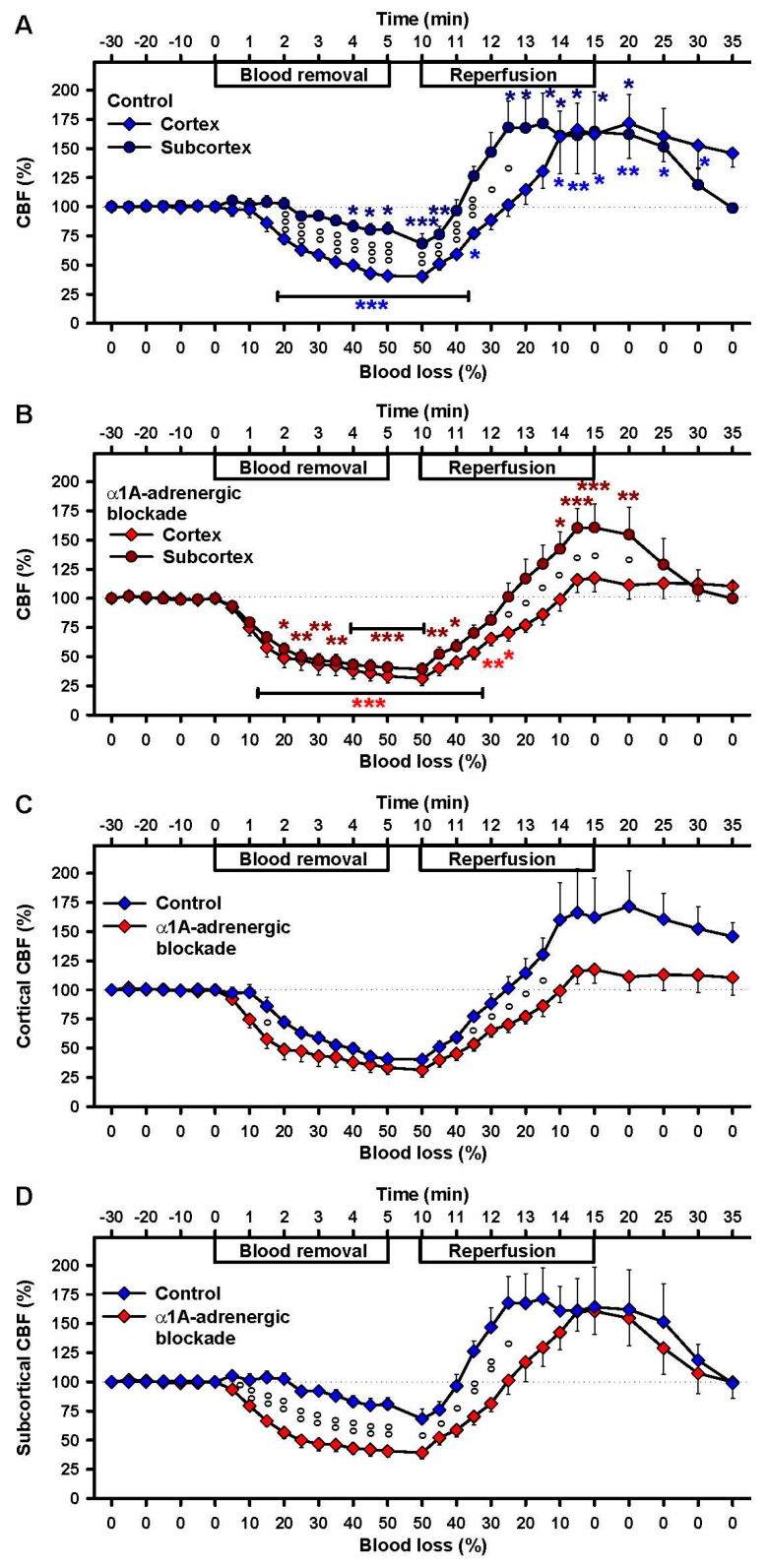
Effects of 50% blood loss and reperfusion on cortical and subcortical cerebral blood flow (CBF). Comparison of cortical and subcortical CBF in the control group (**A**) and under α1A-adrenergic blockade (**B**). Comparison of controls and α1A-adrenergic blockade for (**C**) cortex and (**D**) subcortex. Means ± SEM; * *p* < 0.05, ** *p* < 0.01 and *** *p* < 0.001 compared to baseline; ° *p* < 0.05, °° *p* < 0.01 and °°° *p* < 0.001 for comparison between two experimental groups.

Cortical CBF remained constant until a blood loss of 10%. Subsequently it decreased to 40 ± 4% of baseline at a blood loss of 50% (*p* < 0.001, Figure 3A). In contrast, subcortical CBF remained constant until a blood loss of 20% and subsequently decreased to 81 ± 6% of baseline at a blood loss of 50% (*p* < 0.001, Figure 3A). The slope of the cortical CBF decrease was more than three-fold higher than the slope of the subcortical CBF decrease (calculated by a linear model; 6.3% versus 1.7% loss of blood flow per min over 10 min after start of blood removal; *p* < 0.001).

Reperfusion of 10% blood was sufficient to restore subcortical CBF to baseline, whereas 25% blood reperfusion was needed to reach baseline levels for cortical CBF. Complete replenishment of blood volume led to significantly elevated cortical CBF, reaching a maximum of 172 ± 30% (Figure 3A). Subcortical CBF increased faster, reaching a maximum of 171 ± 26% (Figure 3A) and fell back to baseline 20 min after restoration of the initial blood volume. The important finding of this study is that the responses of cortex and subcortex to blood loss started to differ significantly when 20% or more blood were lacking (Figure 3A).

### 2.3. Correlation of Blood Flow and MABP

**Figure 4:**
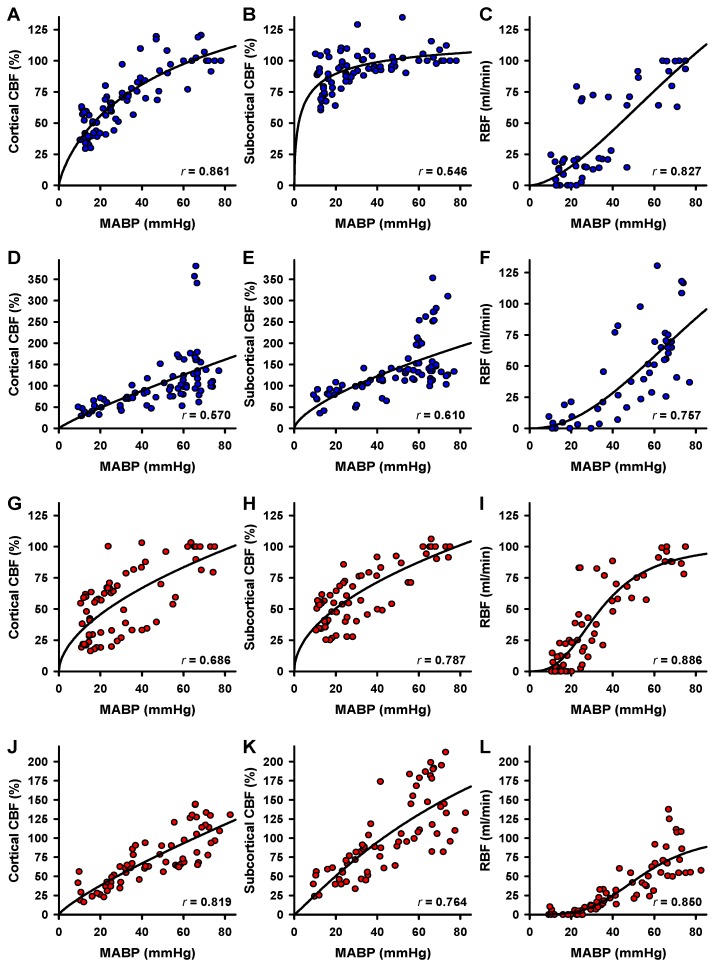
Correlation of blood flow and MABP. Cortical or subcortical CBF or RBF in the control group (blue symbols) were plotted against MABP during blood withdrawal (**A**–**C**) and for the reperfusion phase (**D**–**F**), respectively. The effect of α1A-adrenergic blockade (red symbols) on the relationships of blood flow and MABP during blood withdrawal (**G**–**I**) or during reperfusion (**J**–**L**) is plotted analogously. Three-parameter logistic regression was calculated for each data set (solid lines). Correlation coefficients (*r*) are given in the respective panels, *p* < 0.00001 for all data sets.

To test the relationship of blood flow and MABP, region-specific blood flow was plotted versus MABP. Data were plotted for the blood removal phase (Figure 4A–C) and for the reperfusion phase (Figure 4D–F) separately. For all data sets (cortical CBF, subcortical CBF or RBF; during blood removal or reperfusion), three-parameter logistic regression curves with highly significant correlations for blood flow and MABP could be fitted to the data (Figure 4; correlation coefficients are given in the respective panels; *p* < 0.00001 for all data sets). While there resulted an almost linear curve for RBF, the curves for both cortical and subcortical CBF indicate redistribution of blood flow (Figure 4A–C). Apparently, redistribution is fully effective only in the subcortex. Nearly linear curve fits were obtained during reperfusion for the three tissues measured (Figure 4D–F).

### 2.4. Effects of α1A-Adrenergic Blockade

Under α1A-adrenergic blockade with urapidil there were no significant alterations measured for pH, pCO_2_, pO_2_ and sO_2_, whereas the decrease in BE and the increase of lactate were similar to the control group (*p* < 0.05, Figure 1E,F) during hemorrhage and reperfusion. The low dosage chosen for α1A-adrenergic blockade did not affect MABP and HR at baseline and during hemorrhage and subsequent reperfusion. RBF was indistinguishable from controls during hemorrhage but in contrast to controls was restored to baseline after reperfusion (Figure 2C).

In the presence of urapidil the decrease of cortical CBF to 31 ± 6% of baseline paralleled the decrease of subcortical CBF to 39 ± 5% (both *p* < 0.001, Figure 3B). Subsequent reperfusion of 40% blood restored cortical CBF to baseline, which was not exceeded significantly over time. In contrast, in the subcortex an equal degree of reperfusion resulted in CBF significantly exceeding baseline (peak at complete reperfusion, 161 ± 20%, *p* < 0.001, Figure 3B). This observation shows for the first time that blood flow in cortex and subcortex is significantly different during reperfusion when α1A-adrenergic receptors are blocked (Figure 3B).

In cortical CBF-responses, the α1A-adrenergic blockade exerts its effect mainly during reperfusion: it prevents the hyper-perfusion observed during restoration of the blood volume (Figure 3C). Interestingly, in the subcortex the CBF-response to blood loss in the presence of urapidil was significantly stronger than in the control group during hemorrhage and reperfusion (Figure 3D). In addition, hyperperfusion was delayed but not prevented during reperfusion.

Under α1A-adrenergic blockade correlation analysis for cortical CBF, subcortical CBF or RBF, respectively, in relation to MABP revealed no difference between the brain regions during hemorrhage (Figure 4G–I; *p* < 0.00001 for all data sets). As seen in the control animals, nearly linear curve fits were obtained during reperfusion for the three tissues measured (Figure 4J–L).

### 2.5. Expression of α1-Adrenergic Receptors

**Figure 5:**
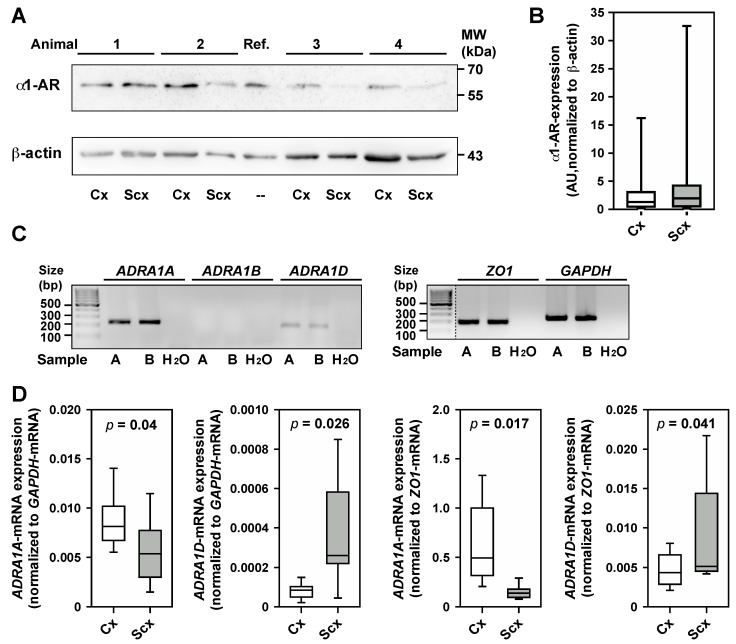
Expression of α1-adrenergic receptors in cortical and subcortical brain arterioles. (**A**) Western blot analysis was performed for cortical and subcortical brain arterioles from 14 sheep, as described in the methods section. α1-adrenergic receptors (α1-AR) are detectable in cortex (Cx) and subcortex (Scx). 1–4, samples from different sheep; AU, arbitrary units; Ref., reference sample; (**B**) Quantification of receptor expression after normalization of band intensities to the housekeeping protein β-actin shows no differences of expression levels in cortex and subcortex, respectively; (**C**) Quantitative PCR-analysis was done using cDNA reverse transcribed from total RNA of 7 sheep. PCR-products of correct size were consistently detected for housekeeping genes *GAPDH* and *ZO-1*, and for the α1-adrenergic receptors A (*ADRA1A*) and D (*ADRA1D*), but not for the B-subtype receptor (*ADRA1B*). A, B, depict examples from two individual sheep; H_2_O, no cDNA control; (**D**) Quantitation of receptor mRNA-expression in relation to the *GAPDH mRNA* and the *ZO-1 mRNA*, respectively, by the ΔΔ*C*_t_-method. As some data sets were not normally distributed all quantitations are presented as box plots, where boxes represent 25th and 75th percentiles, respectively. Medians are indicated by horizontal lines. Whiskers indicate 10th and 90th percentiles, respectively. *p*-values indicating significant differences are given in the panels.

Brain arterioles from the cortex and subcortex showed no difference in the expression of α1-adrenergic receptors (α1-AR) at the protein level (Figure 5A) in both brain regions under investigation (Figure 5B). Quantitative RT-PCR was used to definitively clarify which α1-AR subtypes are expressed in brain arterioles of the cortex and subcortex. In both brain regions mRNA-expression for *ADRA1A* and *ADRA1D* was found, whereas *ADRA1B* was not expressed (Figure 5C). Interestingly, *ADRA1A* expression in cortical arterioles is significantly stronger than in subcortical arterioles, whereas expression of *ADRA1D* is significantly stronger in subcortical arterioles (Figure 5D). This expression pattern was not dependent on the gene used for normalization—a prototypical housekeeping gene (*GAPDH*) or an endothelial marker (*ZO-1*).

## 3. Discussion

In this study, we identified differential regulation of cortical versus subcortical cerebral blood flow (CBF) as a pathophysiological correlate to the greater vulnerability of the cerebral cortex in situations of severe blood loss.

### 3.1. Clinical Implications

Using the sheep model, we were able to continuously measure region-specific CBFs. During controlled hypovolemia, cortical CBF remained constant until a blood loss of 10% compared to subcortical CBF, which remained constant until a blood loss of 20%. Even more important, the rate of reduction of CBF is more than three-fold higher in the cortex as compared to the subcortex, indicating that effective redistribution of blood flow is confined to the latter. It is known that the degree of the decrease of cerebral perfusion is correlated to the severity of brain damage [3]. Taken together, less pronounced redistribution may explain the higher frequency of hypoxic-ischemic brain damage in the cortex as compared to the subcortex of patients with hemorrhagic shock [7,9,10,11,12].

The varying degrees of CBF responses during controlled hemorrhage imply differences in the function of redistribution in the cerebral cortex and subcortex. This does not correspond to the present paradigm that centralization of the blood circulation spares the entire brain during adult life [20,21,22,23].

Indeed, there are some studies, which have shown heterogeneity in CBF in response to severe hemorrhage [13,14,15,16,24,25,26,27]. In summary, using small animal models (rats, rabbits, cats, newborn sheep or pigs) without or with anesthesia, various outcomes were reported. A trend can be delineated: the phylogenetically older subcortex or brainstem regions controlling fundamental functions (e.g., respiration and circulation) seemed to be better protected against hemorrhage (i.e., less decrease, maintenance or even increase of blood flow, depending on the system used). Most of these studies were done either with the iodoantipyrine method or with microspheres, which have the advantage of relatively good spatial resolution. A clear disadvantage of these methods is their lacking suitability for continuous monitoring studies.

Laser Doppler flowmetry allows continuous measurement of CBF [28,29]. Therefore, we used it in the sheep model, which provides more similarities to the human situation than small animals like rodents do [30]. Obviously, studies with a design similar to that which we used, i.e., massive blood removal, cannot be done in human subjects for ethical reasons. However, less severe experimental interventions also indicate that there are different regional CBF responses. In a study with conscious human subjects, Sato et al. described blood flow responses in the internal carotid artery and the vertebral artery using the ultrasound technique for measurements [31]. Most importantly, the reduction in blood pressure caused by a 60-degree head-up tilt resulted in a significant reduction of blood flow in the internal carotid artery, whereas blood flow was essentially unchanged in the vertebral artery. Moreover, they found regional differences in dynamic cerebral autoregulation during a thigh-cuff test [31]. Taken together, despite different experimental settings resulting in different severity of changes of blood pressure, there is clear evidence for region specific redistribution of CBF. In addition, these differences become manifest not only during orthostatic strain but also during extended periods of severe hemorrhage.

In our experimental setup, we tried to limit potential effects of asphyxia. Therefore, blood removal was carried out within 5 min, so that a mere reduction in blood pressure with minimal effects on oxygen supply was ensured. However, even then a reduced pO_2_ with concomitant increase of lactate and decrease of pH reflects a shift to a more anaerobic metabolic condition despite centralization of the circulation. These findings are in agreement with former observations in the same model [32].

The insufficient protection of cortical CBF during hypovolemia may contribute to the higher vulnerability of the cerebral cortex to ischemic brain damage during hemorrhage [7,9]. Our results point out that maintenance of cortical CBF is essential in managing the bleeding patient to improve neurological outcome [6,7,9]. Moreover, our results emphasize that oxygen supply after hemorrhage may protect the brain from hypoxia as ventilation with 100% oxygen during controlled hypovolemia maintained oxygen saturation of the blood.

### 3.2. Potential Roles of α1-Adrenergic Receptors

Hemorrhage is known to induce sympathetic activation leading to contraction of blood vessels in order to counteract the drop in blood pressure. Sympathetic signaling also plays a major role in control of cerebral vascular tone and CBF [33]. Smooth muscle contraction is necessary to maintain or increase vascular tone, and α1-ARs are the most important players in generation of a contractile response (reviewed in [34,35]). The α1-AR subfamily of adrenergic receptors includes three members: α1A, α1B and α1D. Arteries, which exhibit contractile responses to sympathetic stimulation express at least one α1-AR subtype [34,35,36]. However, the contractile response is dependent on various factors, e.g., receptor density, receptor internalization, desensitization, cell specific wiring of signal transduction pathways [34,35,36,37]. All three α1-AR subtypes were shown pharmacologically to be involved in mediation of contractile responses of cerebral artery rings from sheep [38]. The rationale for using urapidil for α1A-adrenergic blockade followed the paradigm that α1A-ARs mediate vasoconstriction after sympathetic activation. This is based on (i) the proven ability of urapidil to lower arterial blood pressure [39]; (ii) the selective inhibition of α1A-ARs by urapidil [35,37]; and (iii) the proven role (by gene knock-out) of α1A-AR in maintenance of arterial blood pressure [40]. Furthermore it has been shown previously that urapidil decreases vascular resistance in all tissues without affecting cardiac output [10] and does not affect baseline CBF [11,12]. From that, we hypothesized that cortical CBF might be preserved with urapidil treatment. To avoid interference by peripheral vasodilatation, we used a dosage, which did not modulate baseline MABP.

Subcortical CBF, but not cortical CBF, is largely maintained during controlled hypovolemia. At first glance this fits nicely with the higher expression levels of α1A-ARs-mRNA in cortical arterioles and the generally accepted role of α1A-ARs to mediate vasoconstriction. But in fact the comparison of cortical CBF without and with α1A-adrenergic blockade suggests that α1A-AR-signaling is necessary for the compensatory mechanism responsible for hyperperfusion when the blood volume is replenished. Furthermore, the redistribution capacity of the subcortex is severely blunted without sufficient α1A-AR-signaling. This indicates that α1A-AR-signaling provides at least a permissive signal for redistribution of CBF—but does not exclude a role in vasoconstriction either.

The observed effects of urapidil are also not consistent with the substance’s known sympatholytic effect, which is mediated by central serotonin 5-HT1A receptors [39]. This effect should have manifested during the baseline measurements. In addition, a direct serotonergic effect of urapidil within the brain vessels also does not explain the prevention of maintenance of subcortical CBF, since the density of 5-HT1A receptors is lower in the thalamus than in the cerebral cortex [41,42,43].

Interestingly, part of the hyperperfusion during reperfusion of the subcortex is refractory to α1A-AR-blockade. We can only speculate, presently, whether the stronger expression of α1D-ARs or the weaker expression of α1A-ARs in the subcortex, which we found at the level of mRNA-expression, may be responsible for the (higher) capacity for redistribution towards the subcortex.

Taken together, our physiological and expression data strongly suggest that in (at least part of) brain arterioles, the role of α1-ARs does not fit the present paradigm of a strictly vasoconstrictory function. This is not without precedent, as at least for the rat carotid artery it was shown previously that functional cross-talk with β-ARs can result in α1D-AR-mediated relaxation [44,45]. Therefore, it will be necessary to further define the receptors involved in redistribution to the subcortex with selective antagonists against various adrenergic receptors in future work.

Our assessment of α1-AR-expression at the protein level was hampered by the lack of subtype-specific antibodies, which recognize the sheep receptors. Therefore, we used only the anti-human pan-specific α1-AR antibody (see methods). The molecular weight of approximately 60 kDa for the diffuse band detected by this antibody corresponds to the full-length α1D receptor or to glycosylated α1A receptor isoforms, thus allowing no discrimination between the receptor subtypes. Consequently the notion of differential expression of α1A-AR and α1D-AR in cortex and subcortex, respectively, is based on mRNA-quantification only. The normalization of expression levels to the endothelial cell marker ZO-1 allows the conclusion that, indeed, vascular α1-AR content differs between cortex and subcortex. As mentioned above, this differential receptor expression might indeed be responsible for the differential effects of hypovolemia on region-specific CBF. Whereas all α1-AR can activate several signal transduction pathways the potency of doing so varies widely [35,36,37]. The increase of intracellular Ca^2+^, which is a prerequisite of smooth muscle contraction necessary for vasoconstriction, is largest upon stimulation of the α1A-AR [36,46]. In contrast, the α1D-AR has the highest potential for activation of mitogen-activated protein kinase pathways [36,47]. Therefore, it is likely—and should be investigated in future studies—that differential activation of downstream signaling pathways is the key for elucidation of the molecular mechanisms underlying the differential regulation of cortical and subcortical CBF under conditions simulating severe shock.

## 4. Materials and Methods

### 4.1. Surgical Instrumentation

All procedures were approved by the Thuringia Animal Welfare Committee (Bad Langensalza; permission number: TVA 02-60/10; valid from 20 December 2010 until 20 December 2015) and conducted in accordance to the ARRIVE guidelines [48]. Thirteen female Merino-long wool sheep at 2–6 years of age weighing 94.5 ± 9.2 kg were included in this study and underwent surgery. After food withdrawal for 24 h, anesthesia was induced by intramuscular injection of 10–15 mg·kg^−1^ ketamine (Ketamin-Hydrochlorid^®^, Pfizer, Berlin, Germany) and 0.2 mg·kg^−1^ midazolam (Midazolam-Hameln^®^, Hameln Pharmaceuticals, Hameln, Germany). After orotracheal intubation anesthesia was maintained by inhalation of 1.5% isoflurane (Isofluran–Actavis^®^, Actavis, Langenfeld, Germany) in 100% oxygen over the entire experiment. All ewes were instrumented with vascular catheters (Arterial Leadercath, Vygon, Aachen, Germany) inserted into the carotid artery for blood sampling and blood pressure measurement and into the jugular vein (Trilyse Expert^®^, Vygon, Lansdale, PA, USA) for intraoperative administration of analgesics and removal of blood. Arterial blood pressure was recorded using transducers (Combitrans Transducer, Braun, Melsungen, Germany). Skin and epicranial aponeurosis were removed from the skull and a borehole trepanation of 1 cm in diameter was performed 4 cm in front of the interauricular line and 1 cm lateral of the midline. Single fiber laser Doppler flow probes (diameter 400 µm, Moor, Devon, UK) were inserted into the brain 2 mm to reach the parietal cortex and 2.7 cm to reach the subcortex (thalamus), respectively, for continuous monitoring of capillary CBF changes. Laser Doppler flowmetry is a reliable tool to assess dynamic changes in capillary cerebral perfusion continuously using the Doppler effect induced by moving red cells [49]. After a left lateral laparotomy we inserted a flow probe (Animal Blood Flowmeter T 206, Transonic, Ithaca, NY, USA) around the left kidney artery to determine renal blood flow (RBF) as a measure of the effects of blood loss on tissues severely affected by major hemorrhage. Electrocardiogram (ECG) was derived using intracutaneous wire electrodes. After the experiment animals were euthanized by intravenous injection of pentobarbital sodium (Narcoren, Merial, Halbergmoos, Germany).

### 4.2. Hypovolemia and Reperfusion

The experimental protocol consisted of 30 min baseline, 5 min controlled hemorrhage, 5 min lag time, 5 min reperfusion and 20 min recovery. After 30 min of baseline recordings, controlled severe hemorrhage was induced in seven sheep by withdrawal of 50% of the estimated total blood volume, which approximates 7% of total body weight in sheep [3,41]. Blood was removed continuously at a rate aiming to remove the estimated blood volume within five min. The blood was saved in heparinized (1000 I.E. Heparin-Natrium, Ratiopharm, Ulm, Germany) empty infusion bags and kept continuously at 37 °C in a water bath. Five min after the withdrawal of 50% of the estimated total blood volume the total blood volume was replenished within five min and blood flow changes were monitored for an additional 20 min. Cortical and subcortical CBF, MABP, ECG, HR, body temperature and oxygen saturation were recorded continuously during hypovolemia and reperfusion. Blood gas samples were taken before the start of the experimental procedure, at 50% blood loss and at the end of the experiments.

### 4.3. α1A-Adrenergic Blockade

The same experimental procedure was performed in six sheep under α1A-adrenergic receptor blockade with urapidil (Urapidil-Phamore^®^, Phamore, Ibbenbüren, Germany). An initial bolus of 8 mg urapidil was injected after 10 min of baseline recordings, followed by continuous infusion at a rate of 8 mg/h over the remaining observation period. This dose was the maximal dose that did not affect MABP, HR and RBF in preliminary experiments.

### 4.4. Sample Preparation and Western Blotting

Brain arterioles from cortex and subcortex were taken after euthanasia from 14 age-matched control sheep that did not undergo the experimental procedure. 3rd branches of region specific arterioles were snap frozen in liquid nitrogen. Sample preparation and Western blotting protocol were described previously [30]. Briefly, an affinity-purified antibody raised against a peptide conserved in all human and sheep α1-adrenergic receptors (α1-ARs) was used for detection of proteins (rabbit anti-pan-α1-AR; 1:1000; Acris Antibodies, Herford, Germany). Mouse anti-β-actin (1:5.000; AC-15, A5441; Sigma-Aldrich, Taufkirchen, Germany) was used for normalization. Secondary antibodies were goat anti-rabbit-IgG-HRP (1:5.000; sc-2004) and goat anti-mouse-IgG-HRP (1:5.000; sc-2031) (all from Santa Cruz Biotechnology, Dallas, TX, USA). For the comparison of α1-AR expression in different gels, a reference sample was included in each gel (produced by mixing nine brain vessel extracts).

### 4.5. Quantitative RT-PCR

Total RNA was extracted from cortex and thalamus of 8 sheep using NucleoSpin RNA (Macherey-Nagel, Düren, Germany), and reverse transcribed into cDNA using the High-Capacity cDNA Reverse Transcription Kit (AppliedBiosystems, Darmstadt, Germany). qRT-PCR was performed in a StepOnePlus cycler (Applied Biosystems) using GoTaq qPCR Master Mix (Promega, Mannheim, Germany), and the following primers (Gene symbol, forward primer, reverse primer): *ADRA1A*, 5′-ACTACATCGTCAACCTGGCG-3′, 5′-GGTAGCGCAGAGGATAGCTC-3′; *ADRA1B*, 5′-CCTTCAAGCTCTTGCCCGA-3′, 5′-CCAGGGGCATGTTGCTTTG-3′; *ADRA1D*, 5′-CATTGTCGTGGGCGTCTTTG-3′, 5′-TGTTGAAGTAGCCCAGCCAG-3′; *GAPDH*, 5′-GAAGGTCGGAGTGAACGGAT-3′, 5′-GATGACGAGCTTCCCGTTCT-3′; and *ZO1*, 5′-CTCCAGGCCCTTACCTTTCG-3′, 5′-CTCGTAAAGAGTCGGCGTGT-3′. Relative expression levels of α1-adrenergic receptor mRNAs (*ADRA1A*, *ADRA1B*, *ADRA1D*) were analyzed using the ∆∆Ct method, and normalized to either *GAPDH* as a marker for total cell mass or *ZO1* as endothelial marker.

### 4.6. Data Analysis

Blood gases and lactate were measured on a standard clinical blood gas analyzer (ABL 600, Radiometer GmbH, Willich, Germany). CBF was recorded using a laser Doppler flowmeter (DRT4, Moor, Devon, UK) as described previously [49]. CBF values are given in arbitrary units. All biophysical variables were amplified and sampled at 1000 Hz using a data acquisition and analysis system (Labchart Pro7, ADInstruments, Spechbach, Germany). MABP was calculated and HR was triggered from R waves continuously. Then, all parameters were averaged over five seconds.

### 4.7. Statistical Analysis

Descriptive statistics (means ± SEM) were used to summarize the outcome parameters of the different measurements. Differences of outcome parameters versus the average of 30 min of baseline were identified using one way repeated measures ANOVA and Holm-Sidak multiple comparisons versus baseline. Pairwise comparisons were done with Student’s *t*-test, or the paired *t*-test, as appropriate. The non-linear dependences of blood flow and blood pressure were fitted using three-parameter logistic regression. All statistical analyses were done with SigmaPlot 13.0 (Systat Software, Erkrath, Germany). *p*-Values of less than 0.05 were considered statistically significant.

## 5. Conclusions

In conclusion, subcortical but not cortical cerebral perfusion is maintained during hypovolemic shock. Maintenance of subcortical perfusion is dependent on α1A-AR-signaling, despite its lower expression in subcortical arterioles. Thus, our results suggest that the effectiveness of cerebral redistribution mechanisms under clinical relevant conditions such as during hemorrhagic shock is inferior in the phylogenetically younger cerebral cortex than in the phylogenetically older subcortex. In other words: the essential functions for survival are better protected than the higher brain functions.

## Abbreviations

α1-AR	α1-adrenergic receptor (at the protein level)
CBF	cerebral blood flow
ECG	electrocardiogram
HR	heart rate
MABP	mean arterial blood pressure
qRT-PCR	quantitative real-time polymerase chain-reaction
RBF	renal blood flow
*ADRA1A* (NC_019459.2)	sheep gene for adrenoceptor alpha 1A (GenBank accession number)
*ADRA1B* (NC_019462.2)	sheep gene for adrenoceptor alpha 1B (GenBank accession number)
*ADRA1D* (NC_019470.2)	sheep gene for adrenoceptor alpha 1D (GenBank accession number)
*GAPDH* (NC_019460.2)	sheep gene for glyceraldehyde-3-phosphate dehydrogenase (GenBank accession number)
*ZO1* (NC_019475.2)	sheep gene for tight junction protein 1 (GenBank accession number)
